# Synergistic Properties of Arabinogalactan (AG) and Hyaluronic Acid (HA) Sodium Salt Mixtures

**DOI:** 10.3390/molecules26237246

**Published:** 2021-11-29

**Authors:** Antonia Di Mola, Francesco Ferdinando Summa, Patrizia Oliva, Francesco Lelj, Stefano Remiddi, Ludovica Silvani, Antonio Massa

**Affiliations:** 1 Department of Chemistry and Biology, University of Salerno, Via Giovanni Paolo II, 84084 Fisciano, Italy; toniadimola@libero.it (A.D.M.); fsumma@unisa.it (F.F.S.); poliva@unisa.it (P.O.); 2 La.M.I. and LaSSCAM INSTM Sezione Basilicata, Dipartimento di Chimica, Università della Basilicata, Via dell’Ateneo Lucano 10, 85100 Potenza, Italy; francesco.lelj@unibas.it; 3 Department of Research and Development, MD-Italy, Via Cancelliera 12, Albano Laziale, 00041 Rome, Italy; stefano.remiddi@md-italy.it

**Keywords:** polysaccharides, hyaluronic acid-arabinogalactan mixtures, diffusion ordered spectroscopY (DOSY), solution rheology, mucoadhesive properties

## Abstract

The properties of mixtures of two polysaccharides, arabinogalactan (AG) and hyaluronic acid (HA), were investigated in solution by the measurement of diffusion coefficients *D* of water protons by DOSY (Diffusion Ordered SpectroscopY), by the determination of viscosity and by the investigation of the affinity of a small molecule molecular probe versus AG/HA mixtures in the presence of bovine submaxillary mucin (BSM) by ^1^HNMR spectroscopy. Enhanced mucoadhesive properties, decreased mobility of water and decreased viscosity were observed at the increase of AG/HA ratio and of total concentration of AG. This unusual combination of properties can lead to more effective and long-lasting hydration of certain tissues (inflamed skin, dry eye corneal surface, etc.) and can be useful in the preparation of new formulations of cosmetics and of drug release systems, with the advantage of reducing the viscosity of the solutions.

## 1. Introduction

**Figure 1:**
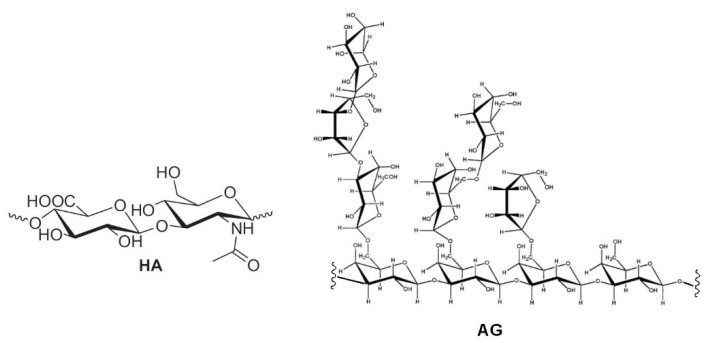
Repetitive unit of hyaluronic acid (HA) and a structural fragment of arabinogalactan (AG).

Hydrogels formed by chemically cross-linked hyaluronic acid (HA) are widely used in the formulation of artificial tears for the treatment of dry eye syndrome [1,2,3,4,5,6,7]. Natural HA is a linear anionic polysaccharide consisting of alternating units of N-acetyl-D-glucosamine and sodium-D-glucuronate groups (Figure 1). With a molecular weight that can reach several millions of Dalton, HA is involved in numerous biological functions [8,9,10,11,12]. It is soluble in water and has a high degree of functionalization and charge density. Its solutions show high viscosity; in solution, it usually arranges in a 3D structure characterized by intramolecular hydrogen bonding [13,14]. This peculiarity also drives its physical–chemical interactions with other molecules. All these properties, as well as its biodegradability and immune neutrality, make HA an optimal biomaterial for wound healing applications and tissue engineering [14,15], in particular, in the regeneration of cartilage [16] and teeth structure [17].

In order to overcome the issues related to chemical cross-linking and modifications of natural HA [5,6,7,8], the development of new formulations, formed by mixtures of different polysaccharides, has been proposed [18,19]. In particular, enhanced mucoadhesive properties have been reported for mixtures of (HA) and tamarind-seed polysaccharide (TSP), a cellulose-like polysaccharide with a high degree of branched glycosyl substitution [18,19]. This mixture is a candidate for new formulations of artificial eye drops. A new artificial eye drop formed by the combination of hyaluronic acid (HA) with another polysaccharide, arabinogalactan (AG), has been recently proposed by Silvani and co-workers [20]. Notably, this mixture synergistically decreases the xanthine oxidoreductase (XOR) activity, inhibiting UA (uric acid) and ROS (reactive oxygen species) formation, and therefore it may contribute to the treatment of the dry eye syndrome, reducing irritation and related pathological conditions. AG is a natural polysaccharide formed of arabinose and galactose in a ratio of 1:6 and a molecular weight ranging between 10,000 and 120,000 Da [21]. AG is mostly extracted from the Larch tree (*Larix occidentalis L. decidua*) but it is abundant in a large variety of plants. Due to the broad array of species, the available AGs show a very wide range of biological properties and documented activities such as the improvement of vascular permeability [22], the support of digestive health by improving intestinal microflora [23,24,25], the enhancement of the immune function [21,22] and a significant in vitro stimulation of dermal fibroblast activity and proliferation [26]. Besides all these properties, AG is also extensively used for treating skin burns and wound-healing in middle and South America [26]. The AG wound-healing property is probably due to the combination of its anti-inflammatory [27], cicatrizing [28] and antimicrobial effects [29]. AG has been approved by the FDA for human consumption in large quantities [30], thus it represents an effective natural alternative to be used for both wound-healing and the reduction of ROS in different matrixes. AG has a high morphological freedom and it has flexible branches with exposed hydroxyl groups (lateral groups) (Figure 1); this peculiarity may play a key role in the interaction with other molecules and polysaccharides.

**Figure 2:**
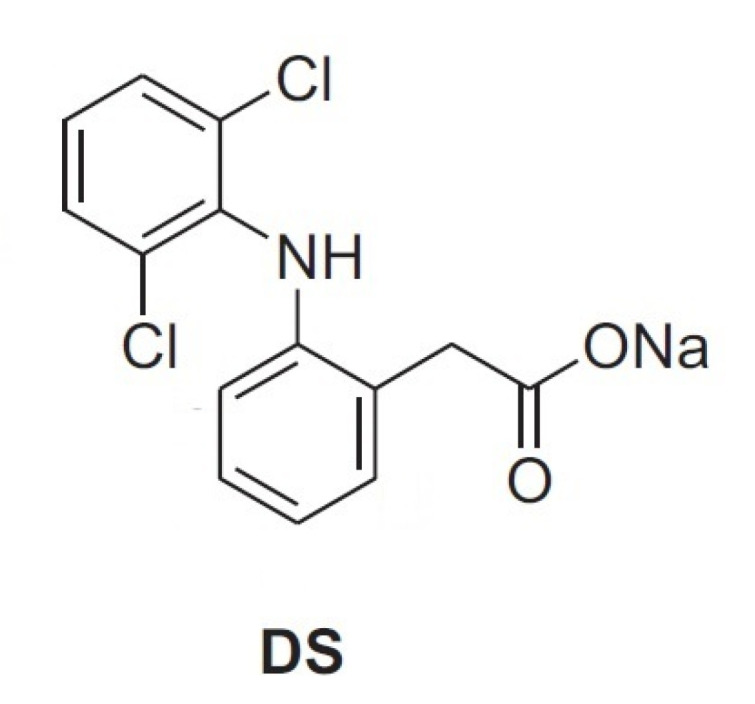
Diclofenac sodium salt.

Nevertheless, to our knowledge, articles reporting physical–chemical properties of mixtures of AG and HA are still lacking. In this context, as part of our interest in the analysis of biomolecules [31], the aim of the present study is the investigation of mixtures of arabinogalactan (AG) and hyaluronic acid (HA) by viscosity measurements and NMR spectroscopy. This investigation can be very useful in highlighting new properties for the development of new formulations of the two polysaccharides for pharmaceuticals and cosmetic applications. The determination of possible synergic effects between different polysaccharides in solution is often challenging and there is a need for simple and reliable methods that can be used to highlight interactions at molecular level. For this purpose, solution rheology and Nuclear Magnetic Resonance (NMR) are particularly useful since the analysis is very simple and reliable, while no sample derivatization or pre-treatment is required [7,12,18,19]. As water is the main component of polysaccharide-based eye drops, studying the behavior of solutions at macroscopic and microscopic levels might show correlations between different polysaccharides, or provide information on their physicochemical properties [7,12,18,19]. In particular, DOSY (Diffusion Ordered SpectroscopY) determines the diffusion coefficient of H_2_O among other molecules and, therefore, this measure can be correlated to the water incorporated in the polymers and to the interactions between polymers of different natures [12]. Additional information about interactions between polysaccharides can be obtained using small molecule molecular probes, as reported in a recent investigation about the properties of solutions of HA and TSP [18,19]. In particular, the formation of stable supramolecular aggregates and enhanced mucoadhesive properties of polysaccharide mixtures have been correlated, by NMR spectroscopic methods in a four-component solution, to their affinity with the anti-inflammatory drug diclofenac sodium salt (DS, Figure 2), a small molecule molecular probe, with respect to bovine submaxillary mucin (BSM) [18,19]. In this case, the interaction between the two polysaccharides was sufficiently effective to perturb the known interaction between DS and mucin and, consequently, the NMR signals of the molecular probe itself.

## 2. Materials and Methods

### 2.1. Chemicals and Materials

Diclofenac sodium (DS) salt, hyaluronic acid, MW 950 kDa (HA), bovine submaxillary mucin (BSM) type I-S and D_2_O 99.9% were purchased from Merck. Arabinogalactan (AG) 20 kDa was purchased from Laracare. The NMR spectra were recorded on Bruker DRX 600 and 400 MHz spectrometers. Viscosity measurements were carried out using a Discovery Hybrid Rheometer HR-2, equipped with coaxial cylinders at shear rates ranging from 0 to 1000 s^−^^1^ (accuracy equal to 1.2%).

### 2.2. Samples Preparation

The stock solutions of each polysaccharide were stirred in a vortex (1000 rpm) for 2 h at 25 °C; after a further 2 h these solutions were employed in the preparation of the binary mixtures for the measurements of diffusion coefficient *D* as well as, in ternary and quaternary mixtures, for the ^1^HNMR studies in the presence of diclofenac sodium salt (DS). All the mixtures were stirred in a vortex (1000 rpm) at 25 °C for 14 h and then transferred into NMR tubes. In all the samples, [DS] = 2.0 mM and mucin BSM = 5 mg/mL. The used quantities of AG and HA are given in the next sections. The same sample preparation was used for the viscosity tests. Viscosity was carried out using a Discovery Hybrid Rheometer HR-2.

### 2.3. Methods

Molecules in liquid or solution state are subjected to translational dynamics. This translational motion is, in contrast to rotational motion, known as Brownian molecular motion and is often simply called diffusion, or self-diffusion, when the chemical potential gradient equals zero. It depends on different macroscopic physical parameters such as: temperature; viscosity; chemical composition; microscopic ones such as size and shape of the molecule; specific interaction within the solution. In general, diffusion is a complex phenomenon which needs a more general description which can be done by a tensor, i.e., a 3 × 3 matrix that considers that it is not necessarily the drag within the medium that gives rise to a motion in the same direction of the drag. This is particularly true in the case of anisotropic medium. Assuming a spherical size of the molecule and isotropic medium, the diffusion motion behavior can be described by a scalar diffusion coefficient *D* within the Stokes–Einstein Equation (1):

(1) $$D=\frac{k\;T}{6\;\pi \;\eta \;r_{s}}$$

where *k* is the Boltzmann constant, *T* the temperature, *η* the viscosity of the liquid and $\;r_{s}$ the hydrodynamic radius of the molecule. According to the IUPAC definition [32], the self-diffusion coefficient D_i_*, for a molecule *i*, is linked to the diffusion coefficient D i {\displaystyle D_{i}} by Equation (2):

(2) $$D_{i}^{*}=D_{i}\frac{\partial \;\ln\;c_{i}}{\partial \;\ln\;a_{i}}$$

where $a_{i}$ is the activity of the species *i* and $c_{i}$. its concentration.

Pulsed magnetic field gradient NMR spectroscopy can be used to measure the translational diffusion of molecules. By use of a magnetic field gradient, molecules can be spatially labeled, i.e., labelled depending on their position in the sample tube. If they move after this encoding during the following diffusion time, their new position can be decoded by a second one. The measured signal is the integral over the whole sample volume and the NMR signal intensity is attenuated depending on the diffusion time and magnetic gradient parameters (g, δ). This intensity change is described by

(3) $$I\left(g\right)=I_{0}\times {\;e}^{-4\pi^{2}{D\gamma }^{2}g^{2}\delta^{2}\left(\Delta -\frac{\delta }{3}\right)\;\times 10^{4}}$$

where I is the observed intensity, I_0_ the reference intensity, *D* the diffusion coefficient, γ the gyromagnetic ratio of the observed nucleus, g the magnetic gradient strength, δ effective magnetic gradient pulse duration, and Δ the diffusion time. ^1^H NMR measurements were performed by a spectrometer operating at 400 MHz. The temperature was controlled to 25 ± 0.1 °C. Experiments performed on the same samples were run in duplicates. *D* was determined from a bipolar-pair longitudinal-eddy-current delay (BPP-LED) DOSY experiment (Bruker pulse sequence ledbpgp2s) using a diffusion delay (Δ) of 149.9 ms, an effective magnetic gradient pulse duration (δ) of 4 ms, a relaxation delay of 20 s and a gyromagnetic ratio of 4258.0 Hz/G for hydrogen atoms. The g value was varied in 16 steps from 2.4 to 45.7 G/cm (scaled to rectangular gradients), with 16 scans acquired for each step. Topspin 4.0 (Bruker) was used for processing and the calculation of diffusion coefficients of water in different samples. Integrals of the water signal were fitted by using Equation (3). The decay curves of $I\left(g\right)/I_{0}$ were checked, to obtain measurements of *D*, for possible two-component behavior. Mono-component exponential fitting was found to give an excellent fit for most samples and was used on all measurements to avoid overfitting (see Supplementary Materials for details). The water signal always appeared as a symmetric signal without shoulders and there was no indication of distinct water signals. The standard error of *D* from the nonlinear fitting was estimated by an iterative approach, where the parameter of interest was varied to define a 95% confidence interval from the sum of squared residuals. The standard deviation between experiments was generally lower than the error from the fitting procedure. The estimated relative standard errors were on average <5%.

## 3. Results and Discussion

### 3.1. Diffusion Coefficients of Water in AG/HA Solutions

Water proton diffusion coefficients *D* determined by DOSY (Diffusion Ordered SpectroscopY) have been recently utilized by Wende et al. [12] in the characterization of crosslinked HA hydrogels in 90/10 H_2_O/D_2_O solution. Therefore, we thought to investigate the properties of AG/HA mixtures at different ratios, measuring *D* of water protons in H_2_O/D_2_O at 90/10 ratio by DOSY using a 400 MHz spectrometer at 25.0 ± 0.1 °C (Table 1). In the preliminary experiments, we soon found that the value of reference *D* (Entry 1) was in accordance with that reported by Wende [12]. Then, we proceeded to analyse solutions of the two polysaccharides. The value of *D* at 1/1 ratio AG/HA (Entry 2) was close to that of reference *D* of water protons. However, considering the standard errors, a sharp comparison cannot be performed. A further increase of AG concentration shows, however, a clear trend, indicating a progressive decrease in the mobility of water (Table 1, Entries 3–5).

*D* values (Table 1) were obtained by nonlinear fitting of the $I\left(g\right)/I_{0}$ decay curves and checked for possible two-component behavior. As observed by Wende and co-workers [12], mono-component exponential fitting was found to give a good fit for most of the samples and used on all measurements to avoid overfitting. Relatively high standard errors were detected [12], comparable to those obtained by us, but, to the best of our knowledge, an investigation about the effect on *D* accuracy of H_2_O/D_2_O at different ratios has never been reported.

Therefore, we decided to determine diffusion coefficients *D* by DOSY using a 400 MHz spectrometer of H_2_O (0.1%) in large excess of D_2_O (99.9%) of solutions of AG and HA, at the same ratios and concentrations, for comparison. Solutions of solely AG or HA were also analyzed (Table 2). The value of reference *D* of water protons was significantly lower (Table 2, entry 1) than that previously determined (Table 1, entry 1). The presence of a larger amount of D_2_O and rapid proton/deuterium exchange would explain the reduced water mobility and therefore the reduced value of *D*. Since the diffusion coefficient is an average measure of bound and unbound water and the H_2_O/HDO ratio, the reduced mobility of water should be referred mainly to as HDO, which is the main species when an excess of D_2_O is present. The mass effect and increased hydrogen-bond strength cause the observed decrease of *D* with respect to those reported in Table 1 [33].

*D* of water protons (0.1%) in D_2_O (99.9%) at 25.0 ± 0.1 °C was also determined for comparison at 600 MHz spectrometer obtaining a value of 1.714 × 10^−9^ m^2^/s, which is in accord with the one, within the experimental error, found at 400 MHz spectrometer (Table 2, entry 1). Encouraged by these preliminary results, we proceeded with an analysis of mixtures of AG/HA at different ratios. For all the tested mixtures, a lower value of *D* of water protons was detected with respect to reference *D* of water (Entry 1), indicating a progressive reduced mobility of water at the increase of AG concentration. A slight increase of water protons *D* value was detected at the ratio AG/HA = 1/1 (3.0 mg/mL total concentration, Table 2, entry 4) with respect to solutions of solely AG or HA at the same concentration (Table 2, entries 2 and 3), probably indicating that the slightly increased mobility of water could be due to the interactions between the two polysaccharides, and the formed aggregates tend to exclude water molecules which are more mobile. Then, a further increase of the AG/HA ratio and concentration (Table 2), leads to a progressive reduced mobility of water protons as previously determined (Table 1). In all the cases lower standard errors were detected (see Table 1 for comparison). The two series of data in Table 1 and Table 2 clearly indicate that the mobility of water is reduced increasing the concentration of AG. Probably, water molecules are entrapped and complexed by carboxylate and hydroxyl groups mainly via hydrogen bonds and van der Waals interactions, causing a macroscopic retainment of a higher amount of water and a progressive decrease in its mobility. These aspects can be of high importance because they can lead to new formulations for pharmaceuticals and cosmetics applications that show better hydrating power and higher retention of water increasing of AG concentration.

However, the decrease in the diffusion coefficient observed at the increase of the total concentration of AG could be ascribed to eventual increase of viscosity. Viscosity greatly affects diffusion of molecules and the diffusion coefficient *D* determined by DOSY experiments [14,32,34]. The diffusion coefficient *D* should be lower in a more viscous fluid, which is consistent with the idealized Stokes–Einstein equation (Section 2.3). Therefore, the determination of viscosity of D_2_O solutions of the mixture of polysaccharides was performed for a better comprehension of the phenomena herein highlighted (see next section).

### 3.2. Determination of Viscosity on D_2_O 99.9% Solutions

**Figure 3:**
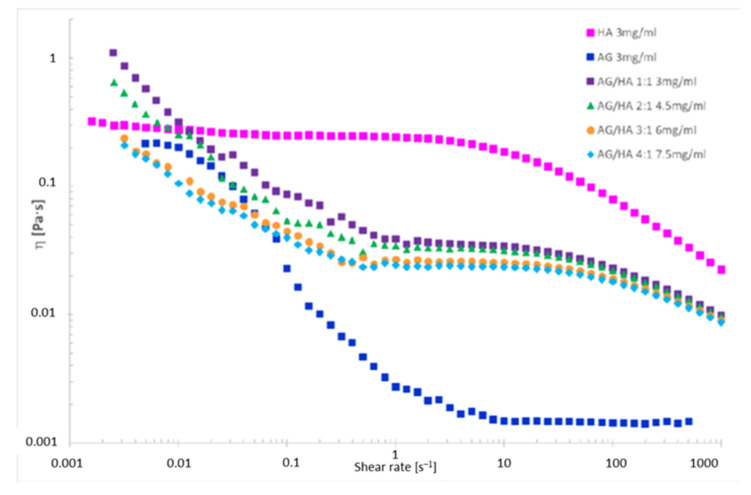
Viscosity vs. the shear rate for polysaccharide solutions in 99.9% D_2_O.

The viscosity was determined on D_2_O solutions of AG/HA mixtures (Figure 1 and Table 3), because D_2_O and H_2_O have different viscosity [35]. The trends of viscosity versus the shear rate for the solely AG, HA and their mixtures is shown in Figure 3. Viscosity values at three different shear rates are listed in Table 3. First of all, the analysis of the curves highlighted an interesting phenomenon, a progressive decrease of the viscosity increasing the total concentration of AG at any shear rate (Figure 3). The correlation of the data in Table 2 with the viscosity measurements (Figure 3) shows lower diffusion coefficients at lower viscosity. This is rather unexpected since the diffusion coefficient *D* should be higher in less viscous fluids, according to the idealized Stokes–Einstein equation (Section 2.3). As reported in the literature, the increase of the molecular weight and concentration of hyaluronic acid in polymer solutions leads to the reinforcement of the three-dimensional network of the polymer, and consequently an increased viscosity [14]. Favored by HA-AG interactions, the decrease of viscosity and the decrease of water mobility observed at the increase of AG concentration can be ascribed to reduction of molecular entanglements and intermolecular interactions between HA chains, and to the strengthening interactions with water. These outcomes have a great practical appeal because an efficient manipulation of the rheological behavior of HA solutions can be achieved when combined with AG. In perspective, these properties can allow a wider use of the two polysaccharides not only in eye-drops preparations, but also as a mix for cosmetics and medical device applications, when decreased viscosity combined with increased hydrating power are required.

The analysis of the viscosity curve of HA solution shows two trends:

1. Shear rate < 1 s^−1^ Newtonian behavior. The entanglements untangle and entangle again, thus viscosity is not affected by the shear rate.

2. Shear rate > 1 s^−1^ pseudoplastic behavior. The entanglements untangle and the chains orient themselves toward the shear direction, the gel deconstructs, the layers become thinner, and the viscosity drops down.

Even though AG in physiological solutions is reported to be a Newtonian fluid [36], the viscosity of the tested AG solution shows that only for a shear rate lower than 0.01 s^−1^ does the polysaccharide seem to display Newtonian behavior, while it drops down at the increase of the shear rate.

In the mixtures herein investigated, AG may behave as non-plasticizing, reinforcing the HA structure. The viscosity decreases with the increasing of the shear rate, because the interactions between macromolecules are weak. Accordingly, higher differences are observed at shear rate < 1 s^−1^ at the increase of AG amount. For high shear rate (>1 s^−1^) the trends are like that of HA, because HA is the only component applying a resistance to the shear. Therefore, the differences in viscosity between the solutions are lower at any used amount of AG and the viscosity trends immediately drop down.

### 3.3. Affinity of the AG/HA Mixtures toward Diclofenac Sodium Salt (DS) vs. Mucin (BSM)

The anti-inflammatory drug diclofenac sodium salt (DS, Figure 2) has been identified as an effective molecular probe to highlight interactions and mucoadhesive properties between HA and tamarind-seed polysaccharide (TSP) in the presence of mucin (BSM) by NMR spectroscopy [18]. Mixtures of TSP and HA were able to perturb the drug–mucin affinity and, therefore, the NMR spectra of the DS. To further investigate the interactions between AG and HA, spectra in D_2_O of pure DS, of binary mixture DS/BSM, ternary mixtures DS/BSM/AG and of quaternary mixtures DS/BSM/AG/HA at different AG/HA ratios and concentrations, have been registered.

**Figure 4:**
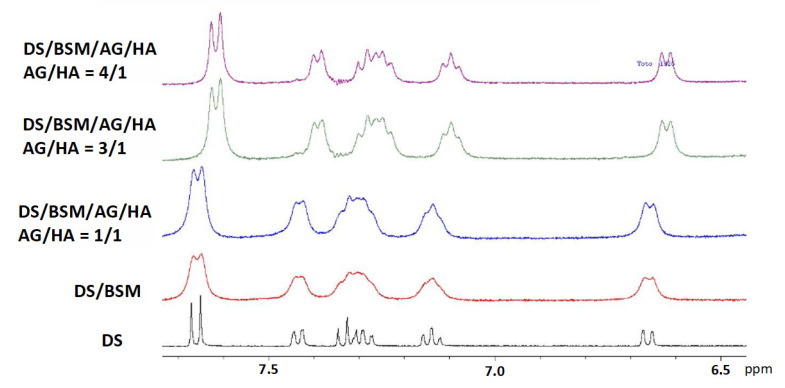
^1^H-NMR spectra in D_2_O 99.9% at 400 MHz in the range 6.5–8 ppm. In all the samples [DS] = 2.0 mM and of mucin BSM = 5 mg/mL. From the bottom: (black line) DS; (red line) binary mixture DS/BSM; quaternary mixtures DS/BSM/AG/HA: (blue line) AG/HA = 1/1, total concentration 3 mg/mL; (green line) AG/HA = 3/1, total concentration 6.0 mg/mL; (purple line) AG/HA = 4/1, total concentration 7.5 mg/mL.

Even though AG in water solution has been reported to show poor mucoadhesive properties [36], comparison of the ^1^H-NMR spectra gave indications regarding the effect of AG/HA ratios on the DS to mucin affinity (Figure 4). In the binary mixture of DS/mucin (red line), as well as in a quaternary mixture with AG/HA = 1/1 (blue line), a remarkable broadening of the resonances of DS was detected, an effect that can be interpreted by the immobilization of DS due to its interaction with mucin [18]. On the other hand, increasing the amount of AG (green and purple line), we observed a progressive improvement of the resolution of the signals, partially recovering the values of the line widths detected for the pure drug (black line). This indicates that, in the mixtures at 3/1 and 4/1 ratios, the molar fraction of free DS is greater than that in the 1/1 mixture [18]. Further increasing the AG amount to 4/1 (purple line), only a slight resolution improvement was observed in comparison to 3/1 ratio. These effects can be explained by the ability of the AG/HA mixture at 3/1 ratio to displace DS from mucin as the consequence of the enhanced mucoadhesive properties of the polysaccharides’ mixture. Similar effects were observed in ^1^H-NMR spectra of DS/BSM/TSP/HA as reported by Uccello-Barretta and co-workers [18], and highlight interactions and mucoadhesive properties between HA and tamarind-seed polysaccharide (TSP).

In addition, a significant variation of the chemical shifts of all the signals of the DS was detected at 3/1 and 4/1 AG/HA ratio with respect to pure DS and to the other analyzed solutions. The interactions with the modified micro-environment led to the modification of the diclofenac chemical shifts and to an incomplete recovery of signal resolution as in a solution of pure DS. Considering that HA is reported not interacting with DS [19], this outcome was further evidence of the effects of AG-HA interactions in solution.

**Figure 5:**
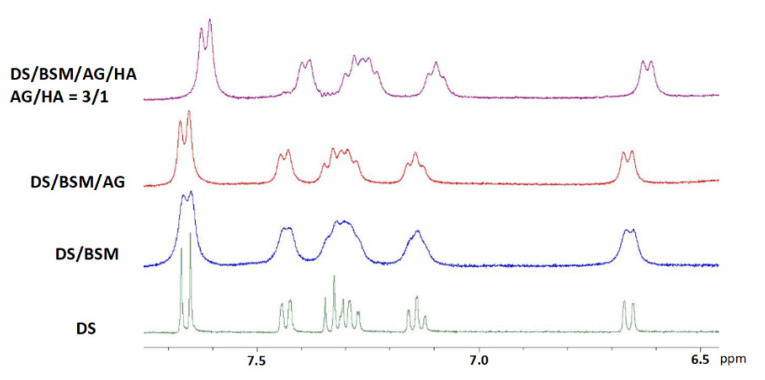
^1^H-NMR spectra in D_2_O 99.9% at 400 MHz in the range 6.5–8 ppm. In all the samples the concentration: [DS] = 2.0 mM, mucin BSM = 5 mg/mL, AG = 3 mg/mL. From the bottom: (green line) DS; (blue line) binary mixture DS/BSM; (red line) ternary mixtures DS/BSM/AG: AG from Laracare at 3.0 mg/mL; (purple line) quaternary mixtures: DS/BSM/AG/HA, AG/HA = 3/1, total concentration 6.0 mg/mL.

For comparison we have also analyzed the tertiary mixtures containing AG (Figure 5), while the analogue mixtures containing HA were neglected since the effect of HA on DS vs. mucin is already described in the literature and it is reported not to interact with HA [19]. The spectrum of ternary mixture (red line) highlights that AG alone (3 mg/mL) is able to improve the resolution of DS signals in comparison with 1/1 mixture of AG/HA (total concentration 3 mg/mL), indicating that DS has a good affinity with AG. However, in these cases we have not detected modifications of DS chemical shifts as for 3/1 (black line) and 4/1 quaternary mixtures.

## 4. Conclusions

In this work the properties of solutions of two polysaccharides, arabinogalactan (AG) and hyaluronic acid (HA), at different AG/HA ratios and concentrations were investigated by viscosity measurements, by the determination of diffusion coefficients *D* by DOSY and by ^1^HNMR analysis using diclofenac sodium salt, (DS) as a small molecule molecular probe. In particular, a significant decrease of the viscosity and a concomitant decrease of water protons diffusion coefficients *D* were detected at the increase of AG concentration, highlighting the interesting property that less viscous solutions show progressively reduced mobility of water. In addition, ^1^HNMR investigations were able to demonstrate enhanced affinity of AG/HA mixture at 3/1 ratio toward the molecular probe DS with respect to mucin, highlighting improved mucoadhesive properties of the mixtures of the two polysaccharides.

In conclusion, mixtures of AG and HA, as herein investigated, are of high interest and can be useful in the formulation of new drug release systems and cosmetics aiming to exert a more effective and long-lasting hydration of certain tissues (inflamed skin, dry eye corneal surface, etc.) with the additional advantage of reducing the viscosity of the solutions and enhanced mucoadhesive properties. This study confirms, by using different methodologies, the virtuous interaction between AG and HA, which is supposed to form stable supramolecular aggregates able to incorporate water molecules that result in less mobility in the end.

## Figures and Tables

**Table 1:** Diffusion coefficients *D* of water protons in D_2_O/H_2_O = 10/90.

Entry	Mixture (Total Conc. Polysaccharides)	Diffusion Coefficient H_2_O (×10^−9^ m^2^/s) ^a^
1	Reference in D_2_O/H_2_O (10/90)	2.07 ± 0.01
2	AG/HA 1:1 (3.0 mg/mL)	2.09 ± 0.02
3	AG/HA 2:1 (4.5 mg/mL)	2.07 ± 0.02
4	AG/HA 3:1 (6.0 mg/mL)	2.04 ± 0.02
5	AG/HA 4:1 (7.5 mg/mL)	1.99 ± 0.01

^a^ Experiments performed on the same samples were run in duplicates.

**Table 2:** Diffusion coefficients *D* of water protons in 99.9% D_2_O.

Entry	Mixture (Total Conc. Carbohydrate)	Diffusion Coefficient H_2_O (×10^−9^ m^2^/s) ^a^
1	Reference in D_2_O 99.9%	1.703 ± 0.007
2	AG (3.0 mg/mL)	1.666 ± 0.005
3	HA (3.0 mg/mL)	1.658 ± 0.005
4	AG/HA 1:1 (3.0 mg/mL)	1.675 ± 0.006
5	AG/HA 2:1 (4.5 mg/mL)	1.666 ± 0.009
6	AG/HA 3:1 (6.0 mg/mL)	1.656 ± 0.005
7	AG/HA 4:1 (7.5 mg/mL)	1.648 ± 0.005

^a^ Experiments performed on the same samples were run in duplicates.

**Table 3:** Viscosity values for three different shear rates.

Sample	η at 0.1 s^−1^ [mPa·s]	η at 1 s^−1^ [mPa·s]	η at 10 s^−1^ [mPa·s]
HA 3 mg/mL	250.14	243.69	186.97
AG 3 mg/mL	22.90	2.73	1.48
AG/HA 1:1 3 mg/mL	86.69	38.87	34.04
AG/HA 2:1 4.5 mg/mL	53.91	34.43	31.66
AG/HA 3:1 6 mg/mL	44.60	26.93	25.48
AG/HA 4:1 7.5 mg/mL	39.81	24.29	23.46

## Data Availability

Not applicable.

## Supplementary Materials

The following are available online: General procedures and selected data about DOSY experiments.

## Sample Availability

Samples of the compounds are not available from the authors.

## References

[1] Johnson M.E., Murphy P.J., Boulton M. (2006). Effectiveness of Sodium Hyaluronate Eyedrops in the Treatment of Dry Eye. Graefes Arch. Clin. Exp. Ophthalmol..

[2] Stuart J.C., Linn J.G. (1985). Dilute sodium hyaluronate (Healon) in the treatment of ocular surface disorders. Ann. Ophthalmol..

[3] Maulvi F.A., Soni T.G., Shah D.O. (2015). Extended release of hyaluronic acid from hydrogel contact lenses for dry eye syndrome. J. Biomater. Sci. Polym. Ed..

[4] Troiano P., Monaco G. (2008). Effect of hypotonic 0.4% hyaluronic acid drops in dry eye patients: A cross-over study. Cornea.

[5] Xue Y., Chen H., Xu C., Yu D., Xu H., Hu Y. (2020). Synthesis of hyaluronic acid hydrogels by crosslinking the mixture of high-molecular-weight hyaluronic acid and low-molecular-weight hyaluronic acid with 1,4-butanediol diglycidyl ether. RSC Adv..

[6] Berkó S., Maroda M., Bodnár M., Eros G., Hartmann P., Szentner K., Szabó-Révész P., Kemény L., Borbély J., Csányi E. (2013). Advantages of cross-linked versus linear hyaluronic acid for semisolid skin delivery systems. Eur. Polym. J..

[7] Barbucci R., Leone G., Chiumiento A., Di Cocco M.E., D’Orazio G., Gianferri R., Delfini M. (2006). Low- and high-resolution nuclear magnetic resonance (NMR) characterization of hyaluronan-based native and sulfated hydrogels. Carbohydr. Res..

[8] Pavicic T., Gauglitz G.G., Lersch P., Schwach-Abdellaoui K., Malle B., Korting H.C., Farwick M. (2011). Efficacy of cream-based novel formulations of hyaluronic acid of different molecular weights in anti-wrinkle treatment. J. Drugs Dermatol. JDD.

[9] Price R.D., Berry M.G., Navsaria H.A. (2007). Hyaluronic acid: The scientific and clinical evidence. J. Plast. Reconstr. Aesthetic Surg..

[10] Teh B.M., Shen Y., Friedland P.L., Atlas M.D., Marano R.J. (2012). A review on the use of hyaluronic acid in tympanic membrane wound healing. Expert Opin. Biol. Ther..

[11] Schanté C.E., Zuber G., Herlin C., Vandamme T.F. (2011). Chemical modifications of hyaluronic acid for the synthesis of derivatives for a broad range of biomedical applications. Carbohydr. Polym..

[12] Wende F.J., Xuea Y., Nestor G., Ohrlund A., Sandstrom C. (2020). Relaxation and diffusion of water protons in BDDE cross-linked hyaluronic acid hydrogels investigated by NMR spectroscopy—Comparison with physicochemical properties. Carbohydr. Polym..

[13] Mitura S., Sionkowska A., Jaiswal A. (2020). Biopolymers for hydrogels in cosmetics. J. Mater. Sci. Mater. Med..

[14] Snetkov P., Zakharova K., Morozkina S., Olekhnovich R., Uspenskaya M. (2020). Hyaluronic Acid: The Influence of Molecular Weight on Structural, Physical, Physico-Chemical, and Degradable Properties of Biopolymer. Polymers.

[15] Abbruzzese L., Rizzo L., Fanelli G., Tedeschi A., Scatena A., Goretti C., Macchiarini S., Piaggesi A. (2009). Effectiveness and safety of a novel gel dressing in the management of neuropathic leg ulcers in diabetic patients: A prospective double-blind randomized trial. Int. J. Low. Extrem. Wounds.

[16] Eftekhari A., Dizaj S.M., Sharifi S., Salatin S., Saadat Y.R., Vahed S.Z., Samiei M., Ardalan M., Rameshrad M., Ahmadian E. (2020). The Use of Nanomaterials in Tissue Engineering for Cartilage Regeneration; Current Approaches and Future Perspectives. Int. J. Mol. Sci..

[17] Ahmadian E., Eftekhari A., Dizaj S.M., Sharifi S., Mokhtarpour M., Nasibova A.N., Khalilov R., Samiei M. (2019). The effect of hyaluronic acid hydrogels on dental pulp stem cells behavior. Int. J. Biol. Macr..

[18] Uccello-Barretta G., Balzano F., Vanni L., Sanso M. (2013). Mucoadhesive properties of tamarind-seed polysaccharide/hyaluronic acid mixtures: A nuclear magnetic resonance spectroscopy investigation. Carbohydr. Polym..

[19] Uccello-Barretta G., Nazzi S., Zambito Y., Di Colo G., Balzano F., Sanso M. (2010). Synergistic interaction between TS-polysaccharide and hyaluronic acid: Implications in the formulation of eye drops. Int. J. Pharm..

[20] Silvani L., Bedei A., De Grazia G., Remiddi S. (2020). Arabinogalactan and hyaluronic acid in ophthalmic solution: Experimental effect on xanthine oxidoreductase complex as key player in ocular inflammation (in vitro study). Exp. Eye Res..

[21] D’Adamo P. (1996). Larch Arabinogalactan is a Novel Immune Modulator. J. Naturop. Med..

[22] Kelly G.S. (1999). Larch arabinogalactan: Clinical relevance of a novel immune-enhancing polysaccharide. Altern. Med. Rev. A J. Clin. Ther..

[23] Vince A.J., McNeil N.I., Wager J.D., Wrong O.M. (1990). The effect of lactulose, pectin, arabinogalactan and cellulose on the production of organic acids and metabolism of ammonia by intestinal bacteria in a faecal incubation system. Br. J. Nutr..

[24] Marzorati M., Verhelst A., Luta G., Sinnott R., Verstraete W., Van de Wiele T., Possemiers S. (2010). In vitro modulation of the human gastrointestinal microbial community by plant-derived polysaccharide-rich dietary supplements. Int. J. Food Microbiol..

[25] Zippel J., Deters A., Hensel A. (2009). Arabinogalactans from Mimosa tenuiflora (Willd.) Poiret bark as active principles for wound-healing properties: Specific enhancement of dermal fibroblast activity and minor influence on HaCaT keratinocytes. J. Ethnopharmacol..

[26] Villarreal M.L., Nicasio P., Alonso-Cortés D. (1991). Effects of Mimosa tenuiflora bark extracts on WI38 and KB human cells in culture. Arch. Investig. Med..

[27] Rivera-Arce E., Chavez-Soto M.A., Herrera-Arellano A., Arzate S., Agüero J., Feria-Romero I.A., Cruz-Gusmann A., Lozoya X. (2007). Therapeutic effectiveness of a Mimosa tenuiflora cortex extract in venous leg ulceration treatment. J. Ethnopharmacol..

[28] Rivera-Arce E., Gattuso M., Alvarado R., Zárate E., Agüero J., Feria I.A., Lozoya X. (2007). Pharmacognostical studies of the plant drug Mimosae tenuiflorae cortex. J. Ethnopharmacol..

[29] Heinrich M., Kuhnt M., Wright C.W., Rimpler H., Phillipson J.D., Schandelmaier A., Warhurst D.C. (1992). Parasitological and microbiological evaluation of Mixe Indian medicinal plants (Mexico). J. Ethnopharmacol..

[30] FDA (2000). Larch arabinogalactan. Altern. Med. Rev..

[31] De Ferra L., Massa A., Di Mola A., Diehl B. (2020). An effective method for the determination of the enantio-purity of L-α-glycerophosphocholine (L-α-GPC). J. Pharm. Biomed. Anal..

[32] (2014). For the Correlation between Diffusion and Self-Diffusion Coefficient See: IUPAC Compendium of Chemical Terminology.

[33] Easteal A.J., Edge V.J., Woolf L.A. (1984). Isotope Effects in Water. Tracer Diffusion Coefficients for H2180 in Ordinary Water. J. Phys. Chem..

[34] Li D., Kagan G., Hopson R., Paul G., Williard P.G. (2009). Formula Weight Prediction by Internal Reference Diffusion-Ordered NMR Spectroscopy (DOSY). J. Am. Chem. Soc..

[35] Karsten W.E., Lai C.-J., Cook P.F. (1995). Inverse Solvent Isotope Effects in the NAD-Malic Enzyme Reaction Are the Result of the Viscosity Difference between D_2_O and H_2_O: Implications for Solvent Isotope Effect Studies. J. Am. Chem. Soc..

[36] Di Colo G., Zambito Y., Zaino C., Sansò M. (2009). Selected polysaccharides at comparison for their mucoadhesiveness and effect on precorneal residence of different drugs in the rabbit model. Drug Dev. Ind. Pharm..

