# Supplementary material for: Synergistic Properties of Arabinogalactan (AG) and Hyaluronic Acid (HA) Sodium Salt Mixtures
Source: Molecules. 2021 Nov 29;26(23):7246. doi: 10.3390/molecules26237246 (PMC8658974; doi:10.3390/molecules26237246)
Supplement: Supplementary file 1 [file molecules-26-07246-s001.zip › molecules-1491046-supplementary.pdf]

Supplementary information

Synergistic properties of mixtures of arabinogalactan (AG) and hyaluronic acid (HA) sodium salt.

Antonia Di Mola,[a,#] Francesco F. Summa,[a,#] Patrizia Oliva,[a] Francesco Lelj,[b] Stefano Remiddi,[c] Ludovica Silvani[c,*] and Antonio Massa[b,*]

[a]Department of Chemistry and Biology, University of Salerno, Via Giovanni Paolo II, 84084 Fisciano (SA),

[b]La.M.I. and LaSSCAM INSTM Sezione Basilicata, Dipartimento di Chimica, Università della Basilicata, Via dell'Ateneo Lucano 10, 85100 Potenza, Italy.

[c]Department of Research and Development, MD-Italy, Via Cancelliera 12, 00041 Albano Laziale, Rome, Italy.
#ADM and FFS equally contributed.

Table of Contents

General procedures and selected data about DOSY experiments...S2-S7
References S7

DIFFUSION EXPERIMENTS WITH DOSY

Molecules in liquid or solution state move. This translational motion is, in contrast to rotational motion, known as Brownian molecular motion and is often simply called diffusion or self-diffusion.[1] It depends on a lot of physical parameters like size and shape of the molecule, temperature, and viscosity. Assuming a spherical size of the molecule the diffusion coefficient D is described by the Stokes-Einstein equation

$$D = \frac{k\,T}{6\,\pi\,\eta\,r_s}$$

where k is the Boltzmann constant, T the temperature, η the viscosity of the liquid and r_s the (hydrodynamic) radius of the molecule. Pulsed field gradient NMR spectroscopy can be used to measure translational diffusion of molecules. By use of a gradient, molecules can be spatially labeled, i.e. marked depending on their position in the sample tube. If they move after this encoding during the following diffusion time, their new position can be decoded by a second gradient. The measured signal is the integral over the whole sample volume and the NMR signal intensity is attenuated depending on the diffusion time and the gradient parameters (g, δ).[2] This intensity change is described by

$$\mathrm{I(g)} = \mathrm{I_0} \cdot\ \mathrm{e}^{-4\pi^2 \mathrm{D}\gamma^2 \mathrm{g}^2 \delta^2 \left(\Delta - \frac{\delta}{3}\right) \times 10^4}$$

An example of a DOSY experiment is the following:

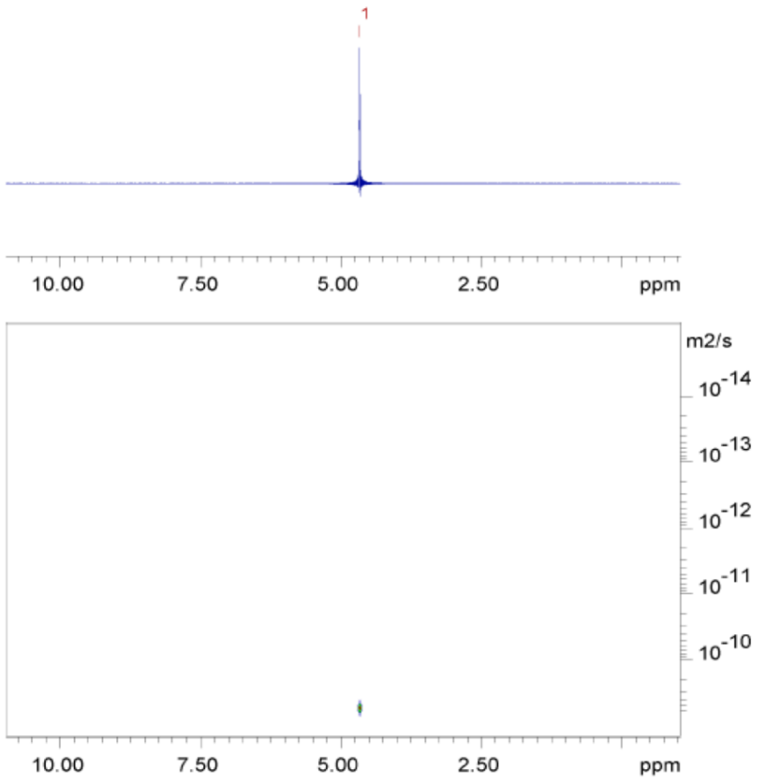

Fitting of DOSY experiments for H_2O in different solutions are given.

H_2O

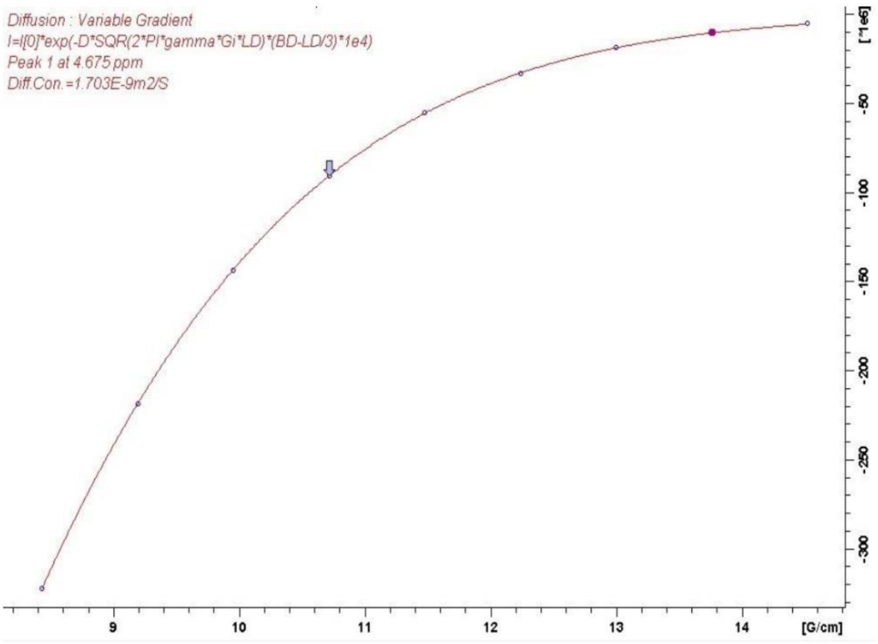

HA 3.0 mg/ml

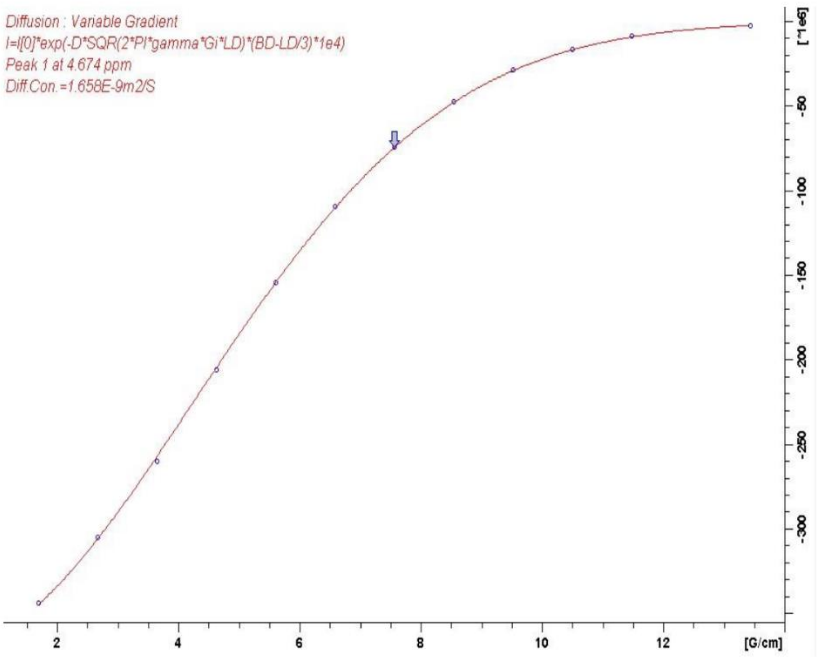

AG 3.0 mg/mL

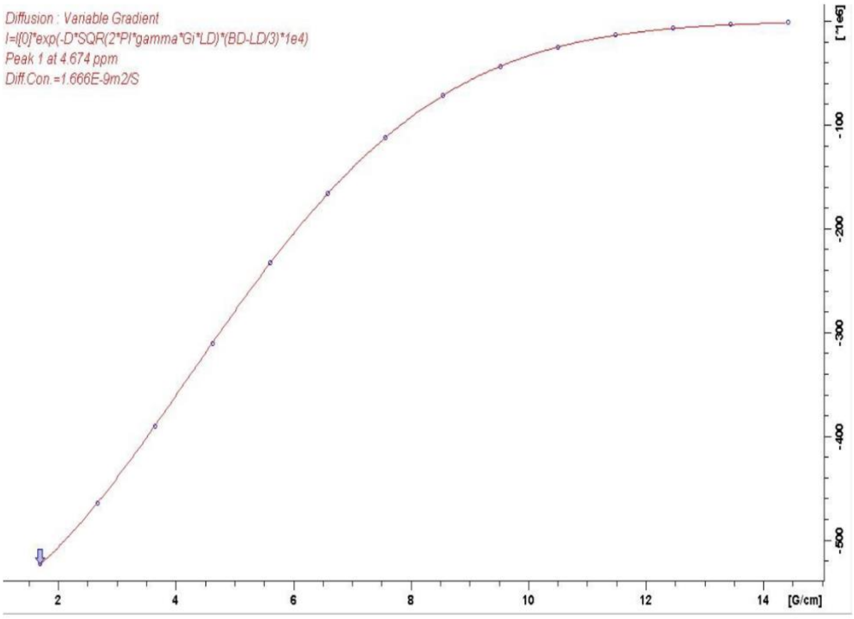

HA/AG 1:1 3.0 mg/mL

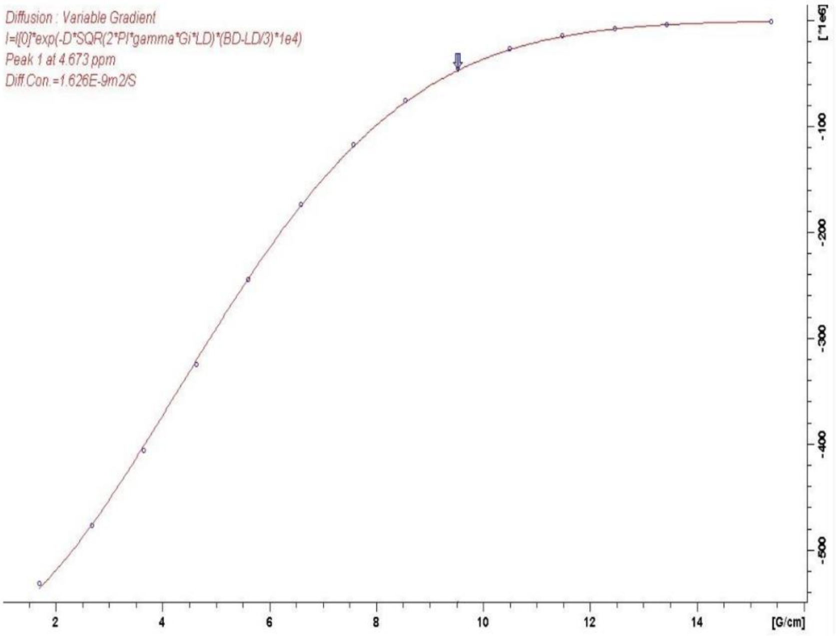

HA/AG 1:2 4.5 mg/mL

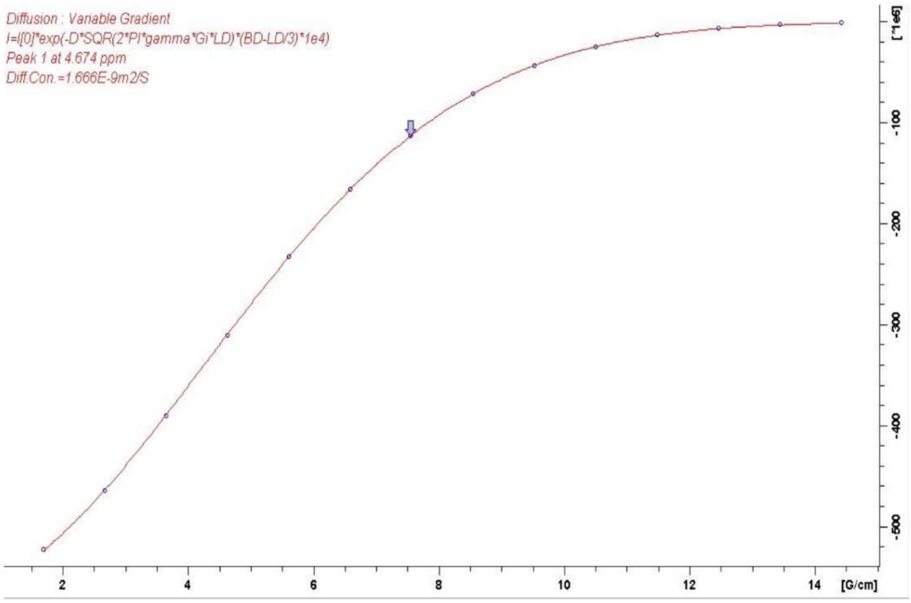

HA/AG 1:3 6.0 mg/mL

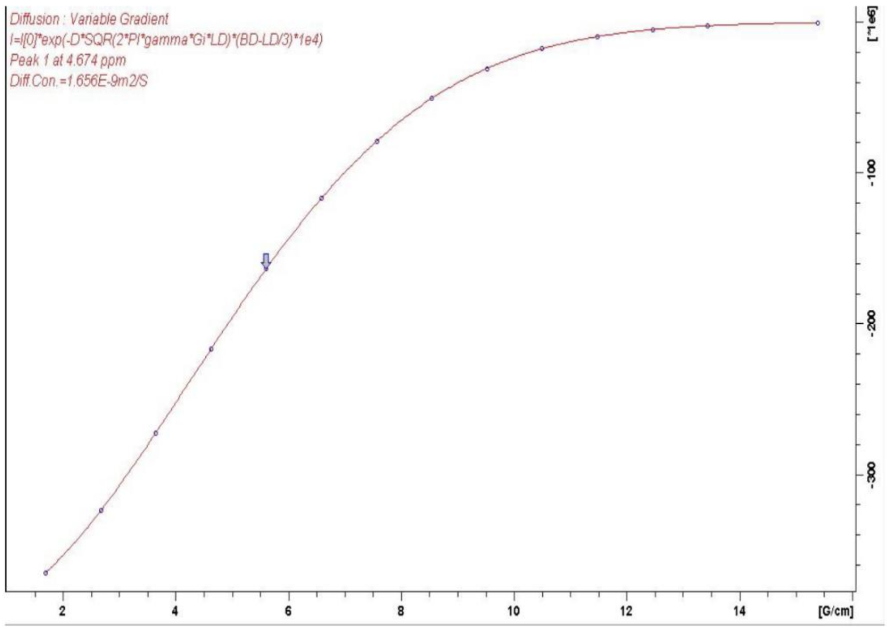

HA/AG 1:4 7.5 mg/mL

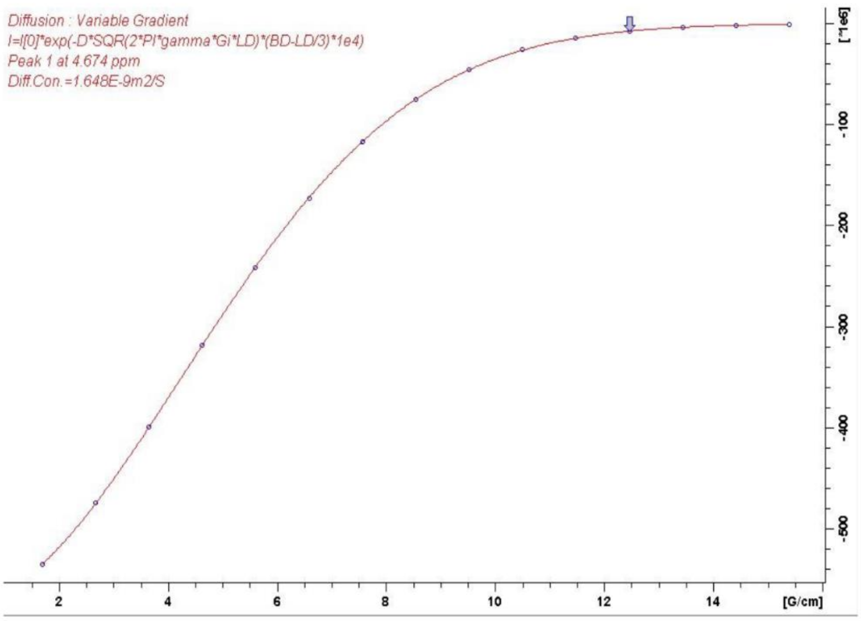

To summarize previous charts, we obtain the following diffusion coefficients:

Table 2. Diffusion coefficients *D* of water protons in 99.9% D_2O

Entry	Mixture (total conc. carbohydrate)	Diffusion coefficient H_2O $(\times 10^{-9} m^2/s)$[a]
1	Reference in D_2O 99.9%	1.703 ± 0.007
2	AG (3.0 mg/mL)	1.666 ± 0.005
3	HA (3.0 mg/mL)	1.658 ± 0.005
4	AG/HA 1:1 (3.0 mg/mL)	1.675 ± 0.006
5	AG/HA 2:1 (4.5 mg/mL)	1.666 ± 0.009
6	AG/HA 3:1 (6.0 mg/mL)	1.656 ± 0.005
7	AG/HA 4:1 (7.5 mg/mL)	1.648 ± 0.005

where the experimental conditions are

T	25°C
γ	4258.0 Hz/G
δ	4.0 ms
Δ	149.9 ms

Also experiments with different conditions have been performed to find the best experimental conditions and to check obtained data. In particular, another experiment was carried out with the 600 MHz instrument at 25°C. From the 600 MHz experiment at 25 °C carried out under the same conditions as the previous ones (400 MHz) we obtain the following chart for the diffusion coefficient of H_2O.

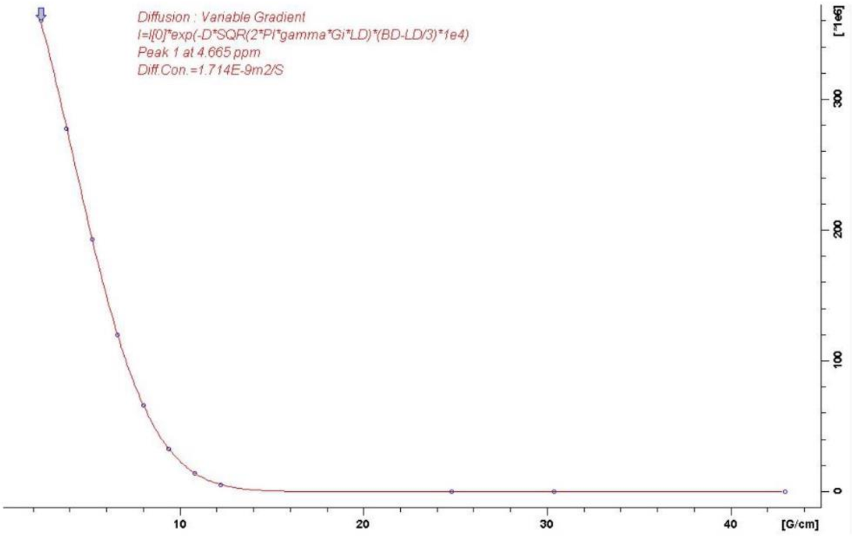

from which we can obtain a diffusion coefficient of 1.714 x10^{-9} m^2/s which is in accord with the previous one, within the experimental error.

References

1. For the correlation between diffusion and self-diffusion coefficient see: IUPAC Compendium of Chemical Terminology, 2nd edition **2014,** https://doi.org/10.1351/goldbook.S05582
2. Wende F. J.; Xuea Y.; Nestor G.; Ohrlund A.; Sandstrom C. Relaxation and diffusion of water protons in BDDE cross-linked hyaluronic acid hydrogels investigated by NMR spectroscopy—Comparison with physicochemical properties. *Carbohydrate Polymers* **2020**, *248*, 116768-116775
